# Impact of chemotherapy cycles and intervals on outcomes of nonspinal Ewing sarcoma in adults: a real-world experience

**DOI:** 10.1186/s12885-019-6407-5

**Published:** 2019-12-02

**Authors:** Jianjun Zhang, Yujing Huang, Yuanjue Sun, Aina He, Yan Zhou, Haiyan Hu, Yang Yao, Zan Shen

**Affiliations:** 1 Department of Oncology, Shanghai Jiao Tong University Affiliated Sixth People’s Hospital, 600 Yishan Rd, Shanghai, 200233 China; 2 Department of Medical Oncology, Shanghai Jiao Tong University Affiliated Sixth People’s Hospital South Campus, 6600 Nanfeng Rd, Shanghai, 201499 China

**Keywords:** Ewing sarcoma, Chemotherapy, Adult, Outcome, Prognostic factor

## Abstract

**Background:**

Adult Ewing sarcoma (ES) is a rare disease, the optimal treatment model is unknown. This study aimed to retrospectively analyze treatment-related prognostic factors of nonspinal ES in Chinese adults.

**Methods:**

Eighty-one patients treated between January 2005 and December 2017 were included in the present study. Thirty-three (40.7%) presented with metastatic disease at diagnosis. Eight patients were submitted to primary surgery followed by chemotherapy, while 73 patients received chemotherapy before and after surgery and/or local radiotherapy. The chemotherapy regimen included 8–17 cycles of vincristine, doxorubicin, and cyclophosphamide (VDC) alternating with ifosfamide and etoposide (IE) every 3 weeks. Clinical outcomes and safety were analyzed.

**Results:**

VDC/IE chemotherapy was well tolerated in adult patients with ES. Multivariate Cox regression analyses revealed that chemotherapy of at least 12 cycles was a favorable independent prognostic factor of event-free survival (hazard ratio, 0.558; 95% confidence interval, 0.323–0.965; *P* = 0.037) and overall survival (hazard ratio, 0.424; 95% confidence interval, 0.240–0.748; *P* = 0.003). Similarly, a low frequency of chemotherapy delays was an independent prognostic factor of improved OS (hazard ratio, 0.438; 95% confidence interval, 0.217–0.887; *P* = 0.022).

**Conclusion:**

Our study suggests that adults with ES should be treated with an aggressive multidisciplinary approach, intensive chemotherapy with adequate cycles and appropriate intervals can be recommended in this group.

## Background

Ewing sarcoma (ES) is the second most common primary bone malignancy in children, but is extremely rare in adults [1–3]. The treatment of ES relies on a multidisciplinary approach that couples risk-adapted chemotherapy and local therapy (surgery, radiation therapy, or both). Chemotherapy plays a pivotal role in the treatment of ES. The regimen including vincristine, doxorubicin, and cyclophosphamide (VDC) alternating with ifosfamide and etoposide (IE) is a category 1 recommendation for ES in NCCN guideline [4]. However, there is no consensus on the optimal chemotherapy cycles and intervals. It was reported that VDC/IE every 3 weeks for 14 cycles contributed to similar survival compared with the same protocol for 17 cycles [5]. VDC/IE given at a 2-week interval was found to be more effective than VDC/IE given at a 3-week interval [5], but the NCCN panel only recommends this 2-week compressed treatment in patients younger than 18 years.

As far as ES in adults is concerned, the optimal chemotherapy regimen remains unknown because current chemotherapy regimens are derived from clinical trials done in a predominantly pediatric population [5–9]. No prospective studies have been performed exclusively in adult ES because of the rarity of the diagnosis. It is not clear whether the pediatric regimens truly apply to adults [10]. The tolerability and efficacy of chemotherapy in older patients should be taken into account when extrapolating the protocols to adults [3, 11]. On the other hand, previous clinical trials in ES were usually conducted in Europe and North America [2, 5–9, 12–14], and were rarely done in Asian countries. There are racial disparities in incidences of ES [15, 16], it is pending whether its response to treatment and prognosis also differ among races. In the present study, we retrospectively reviewed the data of adult ES treated by the same multidisciplinary team over 10 years in China. This study aimed to analyze chemotherapy-related factors that affected outcomes of this rare disease. To our knowledge, this is the largest study on adult ES from Asia. Primary ES of the spine was excluded in our cohort because it has special characteristics and prognostic factors [17].

## Methods

### Patients

This study was approved by the local ethics committee, informed consent was waived due to the retrospective nature of the study. Between January 2005 and December 2017, 87 adult patients with nonspinal ES were treated by the same multidisciplinary team in our center (Table 1). However, 6 patients were excluded either because of insufficient follow-up data or evidence of disease progression before completing first-line therapy. All 81 patients enrolled in the present study had histopathologically diagnosed ES, which met the diagnostic criteria described previously [17]. Evaluation for translocation t (11; 22) (q24; q12) was not required to be enrolled in the study, it was performed in 39 patients. Initial staging procedures consisted of bone marrow biopsies and imaging studies. Imaging studies included X-rays, ultrasonic inspection, computed tomography and magnetic resonance imaging (MRI) of the primary tumor, a chest computed tomography scan, a bone scan or positron emission tomography.

**Table 1:** Demographic and Treatment Characteristics of patients

Characteristics	Number	Percentage
Gender		
Male	56	69.1
Female	25	30.9
Age at diagnosis		
< 30 y	49	60.5
≥ 30 y	32	39.5
Primary site		
Extremity	25	30.9
Trunk	56	69.1
Tumor origin		
Skeletal	57	70.4
Extraskeletal	24	29.6
Stage		
Localized	48	59.3
Metastatic	33	40.7
Diameter of primary tumor		
< 8 cm	50	61.7
≥ 8 cm	31	38.3
Local therapy		
Surgery	35	43.2
Radiotherapy	18	22.2
Surgery + radiotherapy	28	34.6
Number of chemotherapy cycles		
< 12	40	49.4
≥ 12	41	50.6
Frequency of chemotherapy delays		
< 25%	65	80.2
≥25%	16	19.8
Administration of dexrazoxane		
YES	39	48.1
NO	42	51.9

### Treatments

All patients received chemotherapy according to regimen described previously [17]. The regimen included vincristine (1.4 mg/m2 on day 1, maximum 2 mg), doxorubicin (75 mg/m2 on day 1), and cyclophosphamide (1.2 g/m2 on day 1) (VDC) alternating with ifosfamide (1.8 g/m2/day, days 1–5) and etoposide (100 mg/m2/day, days 1–5) (IE) every 3 weeks. When patients had received a cumulative doxorubicin dose of 300 mg/m2, dexrazoxane was administrated for cardioprotective purpose before each dose of doxorubicin at 10:1 dose ratio since 2012. Doxorubicin was replaced by actinomycin D (1.25 mg/m2) after reaching a cumulative dose of 450 mg/m2 or couldn’t been tolerated by patients. Patients had a full blood test on the day prior to the cycle due date and chemotherapy was given if the neutrophil count was greater than 1.5 × 10^9^/L, platelet count greater than 80 × 10^9^/L and biochemical parameters were within normal range. Otherwise, chemotherapy would be delayed until hematological recovery. As a routine, sustained grade 3–4 neutropenia for > 3 days or neutropenic fever was an indication of dose reduction in the present study. Granulocyte colony-stimulating factor prophylaxis was recommended to avoid new episodes of neutropenia and delay of subsequent courses.

The timing and form of local therapy was at the discretion of the multidisciplinary treating team. Whenever possible, surgery was recommended for local treatment. If it was not feasible or not preferred by the patient, definitive radiotherapy would be performed. Indications for postoperative radiotherapy included gross residual disease and positive or close resection margins. The radiotherapy was delivered as previously described [17].

### Data collection

A database was designed to retrospectively collect data on baseline demographic characteristics, treatment modalities, and clinical outcomes. Chemotherapy toxicity was reported according to the Common Terminology Criteria for Adverse Events version 4.0. Chemotherapy given > 3 days later than the cycle due date was defined as a chemotherapy delay. Time to local therapy was defined as the time from the initiation of any treatment to local therapy. If a patient received both surgery and radiotherapy, the date of local therapy was defined by whichever occurred first. Event-free survival (EFS) was defined as the time from diagnosis to disease recurrence, progression, second malignancy, death from any cause, or last contact. Overall survival (OS) was defined as the interval between diagnosis and death from any cause or the most recent follow-up. Clinical responses were classified as complete response (CR), partial response (PR), stable disease (SD), and progressive disease (PD) according to response evaluation criteria in solid tumors for the soft-tissue component of the primary lesion as well as non-osseous metastases [18]. Missing data were collected from patients or their family members by telephone. Information regarding treatment at recurrence was not collected.

### Statistical analysis

All analyses were performed using Statistical Package for the Social Sciences (SPSS), version 18.0 (Chicago, Illinois, USA). Continuous variables were presented as mean ± SD. Qualitative variables were shown as absolute and relative frequencies. The χ^2^ or Fisher’s exact test was used to compare proportions. Kaplan-Meier survival curves were used to estimate proportion surviving and the log-rank test was used to compare differences among subgroups. Cox regression models were used to identify independent prognostic factors. If variables were significant at the 0.1 level on univariate analysis, then they were included in the multiple regression. A *P* value < 0.05 was considered significant.

## Results

### Patient characteristics

This study consisted of 81 patients (56 males, 25 females), with a mean age of 31.0 ± 9.4 years (range 18–63 years) at diagnosis. Forty-nine patients (60.5%) were < 30 years of age, whereas 32 patients (39.5%) were ≥ 30 years. The primary tumors were skeletal in 57 (70.4%) patients, while 24 (29.6%) had extraskeletal disease. Twenty-five patients had extremity-based tumors, including those in the lower (*n* = 17) and upper (*n* = 8) extremities. 56 patients had trunk-based tumors, including those in the pelvic girdle (*n* = 23), in the thorax (*n* = 17), at head and neck (*n* = 9), and in abdomen and hip (*n* = 7). The diameter of primary lesion was < 8 cm in 50 cases and ≥ 8 cm in 31 patients. Among all 81 patients, 48 (59.3%) patients presented with localized disease, 33 (40.7%) patients presented with metastatic disease (14 with lung metastases, 10 with bone metastases, 1 with lymphonode metastases, 7 with both lung and bone metastases, 1 with simultaneous bone and bone marrow metastases) at diagnosis (Table 1).

All patients received chemotherapy, 8 after local treatment and 73 as neoadjuvant and adjuvant therapies. The median number of cycles received per patient was 12 (range, 8 to 17). For the 8 patients who received chemotherapy after local treatment, primary surgery was performed for curative purpose because the treating surgeon did not suspect the presence of ES. Among the remaining 73 patients, 27 patients underwent surgery of the primary tumors as local therapy, 18 patients received radiotherapy alone, and 28 patients received both. Overall, the median time to local therapy was 3.2 months (range, 0–7 months).

### Clinical outcomes

The clinical response in all 73 patients who underwent neoadjuvant chemotherapy included 19 CRs, 37 PRs and 17 SDs, with an overall response rate of 76.7%. Pathologic evaluation of chemotherapy-related tumor necrosis was available for 45 patients who received neoadjuvant chemotherapy; good response (necrosis of ≥90% of the resected specimen) was observed in 20/32 (62.5%) patients with skeletal tumor and in 6/13 (46.2%) patients with extraskeletal disease.

The median follow-up in this cohort was 36 months (range, 11–120 months). Local relapses occurred in 23 patients, 7 of them had undergone exclusive surgery as local therapy, 6 of them had received radiation as local therapy, and the other 8 patients had received combined surgery and radiotherapy, the incidences of local relapses in these three subgroups were similar (*P* > 0.05). Among the 23 patients with local relapses, 10 presented simultaneous metastatic relapses (including metastatic progression) and 13 delayed metastatic relapses. Thirty patients experienced isolated metastatic relapse as a first oncological event. Local and metastatic relapses occurred within a median interval of 23 months (range, 11–49 months). Overall, the 5-year EFS was 22.0%. Fifty-two patients died of disease progression, no death from any other cause occurred, leading to a 5-year OS of 33.0%.

### Safety

Drug toxicities to the hematologic system, liver, kidneys, heart, bladder, mucosa and peripheral nerve were observed during chemotherapy. Grade 3–4 neutropenia was reported in 77 patients (95.1%), grade 3–4 thrombocytopenia in 45 patients (55.6%). Red blood cells or platelets transfusions were needed in 13 (16.0%) and 9 (11.1%) patients, respectively. Peripheral neurologic toxicity occurred in 10 patients; one patient discontinued vincristine early due to grade 3 neurologic toxicity. Toxicity-related dose reduction occurred in 48 patients (59.3%) (Table 2). Forty-two patients (51.8%) had at least one chemotherapy delay, while 16 patients had chemotherapy delays in more than 25% cycles. The major cause of chemotherapy delays was chemotherapy toxicity (87.9%), other reasons accounted for 12.1%. Notably, frequencies of dose reduction were similar in the two patient subgroups who received chemotherapy more or less than 12 cycles (65.9% vs. 52.5%, *P* > 0.05), so the number of chemotherapy cycles positively correlated with the total chemotherapy doses and the frequencies of chemotherapy delays positively correlated with chemotherapy intervals.

**Table 2:** Chemotherapy toxicity in patients

Toxicity and grade	Number	Percentage
Hematological toxicities		
Neutropenia		
All	81	100
3–4	77	95.1
Anemia		
All	29	35.8
3–4	17	21.0
Thrombocytopenia		
All	76	93.8
3–4	45	55.6
Nausea and vomiting		
All	61	75.3
3–4	20	24.7
Hepatic dysfunction		
All	13	16.0
3–4	6	7.4
Renal dysfunction		
1–2	4	4.9
Mucositis		
All	16	19.8
3–4	9	11.1
Cardiac toxicities		
Arrhythmia		
1–2	11	13.6
Myocardial ischemia		
1–2	7	8.6
Hemorrhagic cystitis		
1–2	4	4.9
Neurologic toxicity		
All	10	12.3
3–4	1	1.23
Dose reduction		
All	48	59.3

### Analysis of prognostic factors of survival

**Fig. 1:**
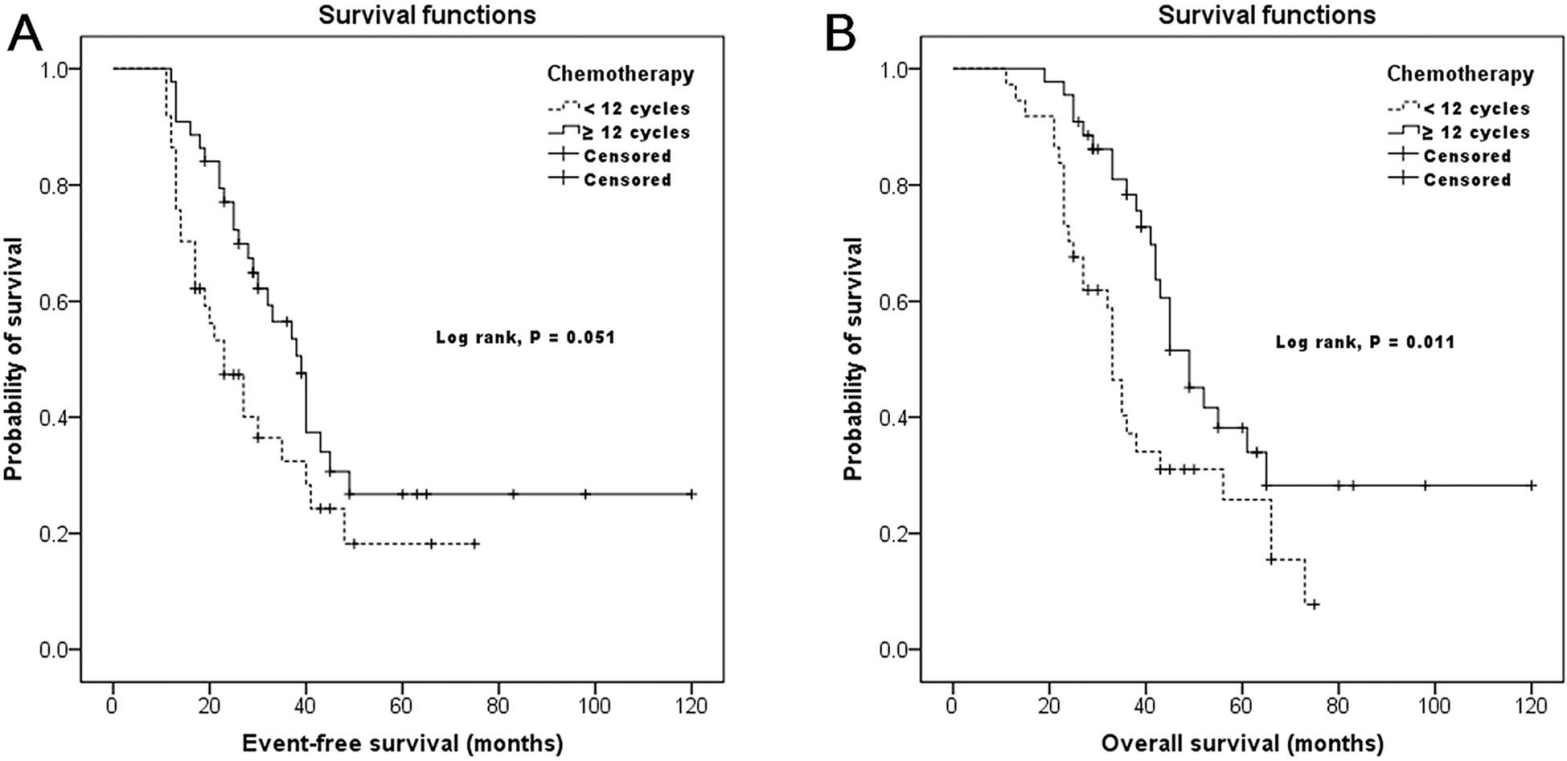
Kaplan-Meier survival curves for event-free survival (**a**) and overall survival (**b**) according to number of chemotherapy cycles

Univariable analysis showed that stage (*P* = 0.071) and number of chemotherapy cycles (*P* = 0.058) showed a trend toward significance for EFS. These two factors were submitted to multivariable analysis. The results showed that both localized disease at diagnosis (*P* = 0.045) and chemotherapy of at least 12 cycles (*P* = 0.037) were favorable independent prognostic factors of EFS (Table 3). As far as OS was concerned, univariable analysis showed that stage (*P* = 0.004) and number of chemotherapy cycles (*P* = 0.015) were significantly associated with OS. These two factors along with frequency of chemotherapy delays (*P* = 0.076) were submitted to multivariable analysis. Multivariable analysis showed that all these three factors were significant predictors of OS (Table 4). Chemotherapy of at least 12 cycles was associated with both improved EFS and OS in the present study (Fig. 1). Stratified analyses indicated that frequency of chemotherapy delays didn’t affect EFS significantly (log-rank test, *P* = 0.364). However, a low frequency of chemotherapy delays (< 25%) was a favorable independent predictor of OS in our patients (*P* = 0.022).

**Table 3:** Univariable and multivariable Cox proportional hazard regression analyses of event-free survival

Factor	Univariable analysis	*P*	Multivariable analysis	*P*
	HR (95%CI)		HR (95% CI)	
Gender				
Male	Reference			
Female	1.107 (0.614–1.993)	0.735		
Age at diagnosis				
< 30y	Reference			
≥ 30 y	1.410(0.820–2.426)	0.215		
Primary site				
Extremity	Reference			
Trunk	0.874 (0.500–1.526)	0.635		
Tumor origin				
Skeletal	Reference			
Extraskeletal	0.771 (0.418–1.421)	0.404		
Stage				
Localized	Reference		Reference	
Metastatic	1.644 (0.958–2.282)	0.071	1.743 (1.012–3.002)	0.045
Diameter of primary tumor				
< 8 cm	Reference			
≥ 8 cm	0.800 (0.458–1.398)	0.434		
Local therapy				
Surgery	Reference			
Radiotherapy	1.504 (0.733–3.087)	0.266		
Surgery + radiotherapy	1.494 (0.806–2.771)	0.202		
Time to local therapy	0.899 (0.732–1.104)	0.309		
Number of chemotherapy cycles				
< 12	Reference		Reference	0.037
≥ 12	0.592 (0.344–1.017)	0.058	0.558 (0.323–0.965)	
Frequency of chemotherapy delays				
≥ 25%	Reference			
< 25%	0.739 (0.380–1.439)	0.374		
Grade 3–4 chemotherapy toxicity				
YES	Reference			
NO	0.382(0.053–2.778)	0.342		
Administration of dexrazoxane				
YES	Reference			
NO	1.057(0.616–1.814)	0.840		

*HR* Hazard ratio, *CI* Confidence interval

**Table 4:** Univariable and multivariable Cox proportional hazard regression analyses of overall survival

Factor	Univariable analysis	*P*	Multivariable analysis	*P*	
	HR (95%CI)		HR (95% CI)		
Gender					
Male	Reference				
Female	1.376 (0.766–2.472)	0.286			
Age at diagnosis					
< 30y	Reference				
≥ 30 y	1.450(0.839–2.507)	0.183			
Primary site					
Extremity	Reference				
Trunk	1.105 (0.620–1.971)	0.735			
Tumor origin					
Skeletal	Reference				
Extraskeletal	0.660 (0.346–1.261)	0.209			
Stage					
Localized	Reference			Reference	
Metastatic	2.269 (1.294–3.976)	0.004		2.488 (1.413–4.383)	0.002
Diameter of primary tumor					
< 8 cm	Reference				
≥ 8 cm	0.765 (0.434–1.351)	0.356			
Local therapy					
Surgery	Reference				
Radiotherapy	1.327 (0.654–2.692)	0.432			
Surgery + radiotherapy	1.271 (0.679–2.379)	0.453			
Time to local therapy	0.901 (0.733–1.107)	0.329			
Number of chemotherapy cycles					
< 12	Reference			Reference	0.003
≥ 12	0.505 (0.291–0.874)	0.015		0.424 (0.240–0.748)	
Frequency of chemotherapy delays					
≥ 25%	Reference			Reference	
< 25%	0.537 (0.270–1.067)	0.076		0.438 (0.217–0.887)	0.022
Grade 3–4 chemotherapy toxicity					
YES	Reference	0.610			
NO	0.595(0.081–4.359)				
Administration of dexrazoxane					
YES	Reference				
NO	0.938(0.540–1.628)	0.820			

*HR* Hazard ratio, *CI* Confidence interval

## Discussion

At present, no standard treatment model has established for adult ES and the management is often institution-specific [19–22]. Even for the most commonly used VDC/IE chemotherapy regimen, the number of cycles ranges from 6 to 17 in different studies [5, 22, 23]. The optimal chemotherapy regimen for adult ES is controversial [10]. Tao et al. reported that there were no significant differences between anthracycline and platinum based chemotherapy regarding EFS and OS [19]. In contrast, Casey et al. and Ahmed et al. reported that treatment according to the pediatric protocol was significantly associated with improved survival [21, 24]. In the present cohort, we also followed the pediatric VDC/IE protocol, unfortunately 50 patients didn’t completed the planned 17 chemotherapy cycles due to various reasons: the most common reason was lack of money (21 patients, 42%), followed by the belief that adults inevitably fare worse than children and increasing chemotherapy duration may not lead to improvement in survival (16 patients, 32%), toxicity-related treatment abandonment occurred in eight patients (16%), and other reasons accounted for five patients (10%). Overall, VDC/IE chemotherapy was well tolerated in our patients. Our study provided real-world data in adult ES from Asian developing countries, and the aforementioned situation provided an opportunity for comparative analysis between different chemotherapy cycles and intervals.

It was reported that adult patients with ES had a worse prognosis compared to pediatric patients [5, 9, 23, 25–27]. As far as nonmetastatic ES was concerned, Granowetter et al. reported that the 5-year EFS was 77.5% for patients age 1 to 9 years, 69.4% for patients age 10 to 17 years, and 63.2% for patients older than 18 years [9]. Womer et al. reported that the 5-year EFS was 72 and 47% for patients younger and older than 18 years, respectively [5]. In the present study, we also observed a poor prognosis for adult ES, with a 5-year OS of 33.0% and EFS of only 22.0%. The reasons behind the poor prognosis for adult ES are unclear. Generally, the prognostic factors of ES include site of primary disease, tumor volume, response to chemotherapy, and presence of metastatic disease at diagnosis [12, 13, 28]. It is not clear if these findings from pediatric studies also apply to adults because several case series suggested that ES in adult populations was a more biologically aggressive variant of the disease [3, 27]. However, there is a uniform consensus that patients presenting with metastatic disease have an extremely poor prognosis, regardless of age. In our cohort, 40.7% of patients presented with metastatic disease at diagnosis, slightly more compared with other series [24, 29, 30], this may partially justify the dismal survival. When patients with metastatic disease at presentation were excluded, the 5-year OS and EFS increased to 44.0 and 31% for the remaining 48 patients in the present study. Notsurprisingly, this outcome was worse than that of aforementioned pediatric patients with localized ES [5, 9]. However, these figures were still fewer than those from other recent adult cohorts: OS of 66% and EFS of 44.0% in the Casey et al. series of 76 cases [24], OS of 66% and EFS of 43.0% in the Fizazi et al. series of 129 cases [30].

To identify the factors that potentially affect the outcomes of our cohort, we conducted exploratory univariable and multivariable analyses. Interestingly, besides metastatic disease at diagnosis, chemotherapy of less than 12 cycles was also detrimental to both OS and EFS of our patients. Moreover, a high frequency of chemotherapy delays (≥ 25%) was an unfavorable independent predictor of OS. As mentioned above, the number of chemotherapy cycles positively correlated with the chemotherapy doses and the frequencies of chemotherapy delays positively correlated with chemotherapy intervals. These findings suggested that the poor prognosis in the present study was partially due to insufficient chemotherapy doses and prolonged dose intervals. Dose-dense chemotherapy with shortening intervals showed an increase in survival of ES [5], whereas the benefit of dose escalation (increasing the dose of chemotherapy agents) studies has been less consistent and may be accompanied by other dose-limiting toxicities [31]. It was reported that lower doses of alkylating agents were detrimental to survival of ES [23], however, dose escalation of alkylating agents in the VDC/IE regimen did not improve the outcome for patients with localized disease [9]. Based on our results, we strongly recommend aggressive treatment for adult ES to maintain adequate chemotherapy doses and appropriate intervals, with the help of supportive treatment. None of the other factors such as extremity/axial primary site, skeletal/extraskeletal tumor origin, diameter of primary tumor, local therapy modality and time to local therapy achieved statistical significance, but age older than 30 years at diagnosis (HR 1.45, *P* = 0.183) showed a trend toward significance for OS. Although chemotherapy cycles and intervals can be affected by chemotherapy toxicity, the univariable analysis indicated that grade 3–4 chemotherapy toxicity didn’t affect the EFS and OS of our patients significantly.

To further explore the potential racial disparity in survival of adult ES, we reviewed the outcomes of localized disease treated with similar cycles of VDC/IE regimen (Table 5). The prognosis of our patients was better than that of Canadian series, while it was a little worse than the prognosis of cohorts from the USA and Turkey. The results showed no evidence of ethnical disparity in survival of adult ES. Further head-to-head comparative studies are needed to clarify this question.

**Table 5:** Clinical outcome of adults with localized Ewing sarcoma

References	Study group	CT regimens	Outcomes
Womer et al. [5]	Prospective study 31 patients ≥18 y (2001–2005, USA)	VDC/IE (including every 2-week protocol) 14 cycles	5-y EFS 47%
Gupta et al. [23]	Retrospective study 24 patients ≥18 y (1990–2005, Canada)	VDC/IE 10 cycles	3-y EFS 43% 3-y OS 59%
Seker et al. [22]	Retrospective study 21 patients ≥19 y (2000–2012, Turkey)	VDC/IE 17 cycles	5-y OS 64%
The present study	Retrospective study 27 patients ≥18 y (2005–2017, China)	VDC/IE ≥ 12 cycles	3-y EFS 60% 3-y OS 81% 5-y EFS 38% 5-y OS 51%

*CT* Chemotherapy, *VDC/IE* Vincristine, doxorubicin, and cyclophosphamide alternating with ifosfamide and etoposide, *EFS* Event-free survival, *OS* Overall survival

Patients who receive high doses of anthracyclines are at risk for cardiotoxicity, dexrazoxane is often used as a cardioprotectant when prior doxorubicin reached a cumulative dose of 300 mg/m^2^ [32]. But controversy exists if dexrazoxane reduces the antitumor effect of doxorubicin and increases the risk of second primary malignancies [33]. In the present study, we found that there were no differences of EFS and OS in patients with or without administration of dexrazoxane (log-rank test, *P* > 0.05), and there was no second primary malignancy occurred during the follow-up period. Our results were consistent with the findings from other recent large population-based studies [32, 34, 35].

This study has several limitations. First, it was a retrospective, single-institution study with a small sample size. As a consequence, it was difficult to draw conclusions on all factors influencing outcomes. Second, we excluded patients for whom there was incomplete clinical data or evidence of disease progression before completing first-line therapy. This could have resulted in selection bias. To assess accurately the outcomes and prognostic factors of adult ES, large prospective clinical trials are needed.

## Conclusion

The present study shows that VDC/IE chemotherapy is well tolerated in adult patients with ES, chemotherapy of at least 12 cycles is associated with better EFS and OS compared with less cycles of chemotherapy. Similarly, a low frequency of chemotherapy delays is an independent prognostic factor of improved OS. Our findings suggest that adults with ES should be treated with an aggressive multidisciplinary approach, intensive chemotherapy with adequate doses and appropriate intervals can be recommended in this group when the toxicity was tolerable.

## Data Availability

The datasets used and/or analyzed during the current study are available from the corresponding author on reasonable request.

## Abbreviations

CR: Complete response
EFS: Event-free survival
ES: Ewing sarcoma
HR: Hazard ratio
IE: Ifosfamide and etoposide
MRI: Magnetic resonance imaging
NCCN: The National Comprehensive Cancer Network
OS: Overall survival
PD: Progressive disease
PR: Partial response
SD: Stable disease
SPSS: Statistical Package for the Social Sciences
VDC: Vincristine, doxorubicin, and cyclophosphamide

## References

[1] Robinson SI, Ahmed SK, Okuno SH, Arndt CA, Rose PS, Laack NN (2014). Clinical outcomes of adult patients with relapsed Ewing sarcoma: a 30-year single-institution experience. Am J Clin Oncol.

[2] Cotterill SJ, Ahrens S, Paulussen M, Jurgens HF, Voute PA, Gadner H (2000). Prognostic factors in Ewing’s tumor of bone: analysis of 975 patients from the European intergroup cooperative Ewing's sarcoma study group. J Clin Oncol.

[3] Ganjoo KN, Patel S (2013). The treatment outcome for adult patients with Ewing's sarcoma. Curr Oncol Rep.

[4] Gaspar N, Hawkins DS, Dirksen U, Lewis IJ, Ferrari S, Le Deley MC (2015). Ewing sarcoma: current management and future approaches through collaboration. J Clin Oncol.

[5] Womer RB, West DC, Krailo MD, Dickman PS, Pawel BR, Grier HE (2012). Randomized controlled trial of interval-compressed chemotherapy for the treatment of localized Ewing sarcoma: a report from the Children's oncology group. J Clin Oncol.

[6] Grier HE, Krailo MD, Tarbell NJ, Link MP, Fryer CJ, Pritchard DJ (2003). Addition of ifosfamide and etoposide to standard chemotherapy for Ewing's sarcoma and primitive neuroectodermal tumor of bone. N Engl J Med.

[7] Burgert EO, Nesbit ME, Garnsey LA, Gehan EA, Herrmann J, Vietti TJ (1990). Multimodal therapy for the management of nonpelvic, localized Ewing's sarcoma of bone: intergroup study IESS-II. J Clin Oncol.

[8] Juergens C, Weston C, Lewis I, Whelan J, Paulussen M, Oberlin O (2006). Safety assessment of intensive induction with vincristine, ifosfamide, doxorubicin, and etoposide (VIDE) in the treatment of Ewing tumors in the EURO-E.W.I.N.G. 99 clinical trial. Pediatr Blood Cancer.

[9] Granowetter L, Womer R, Devidas M, Krailo M, Wang C, Bernstein M (2009). Dose-intensified compared with standard chemotherapy for nonmetastatic Ewing sarcoma family of tumors: a Children's oncology group study. J Clin Oncol.

[10] Wagner MJ, Livingston JA, Patel SR, Benjamin RS (2016). Chemotherapy for bone sarcoma in adults. J Oncol Pract.

[11] Bacci G, Ferrari S, Comandone A, Zanone A, Ruggieri P, Longhi A (2000). Neoadjuvant chemotherapy for Ewing's sarcoma of bone in patients older than thirty-nine years. Acta Oncol.

[12] Rodriguez-Galindo C, Liu T, Krasin MJ, Wu J, Billups CA, Daw NC (2007). Analysis of prognostic factors in Ewing sarcoma family of tumors: review of St. Jude Children’s Research Hospital studies. Cancer.

[13] Orr WS, Denbo JW, Billups CA, Wu J, Navid F, Rao BN (2012). Analysis of prognostic factors in extraosseous Ewing sarcoma family of tumors: review of St. Jude Children's Research Hospital experience. Ann Surg Oncol.

[14] Paulussen M, Ahrens S, Dunst J, Winkelmann W, Exner GU, Kotz R (2001). Localized Ewing tumor of bone: final results of the cooperative Ewing's sarcoma study CESS 86. J Clin Oncol.

[15] Parkin DM, Stiller CA, Nectoux J (1993). International variations in the incidence of childhood bone tumours. Int J Cancer.

[16] Jawad MU, Cheung MC, Min ES, Schneiderbauer MM, Koniaris LG, Scully SP (2009). Ewing sarcoma demonstrates racial disparities in incidence-related and sex-related differences in outcome: an analysis of 1631 cases from the SEER database, 1973-2005. Cancer..

[17] Zhang J, Huang Y, Lu J, He A, Zhou Y, Hu H (2018). Impact of first-line treatment on outcomes of Ewing sarcoma of the spine. Am J Cancer Res.

[18] Ladenstein R, Potschger U, Le Deley MC, Whelan J, Paulussen M, Oberlin O (2010). Primary disseminated multifocal Ewing sarcoma: results of the Euro-EWING 99 trial. J Clin Oncol.

[19] Tao HT, Hu Y, Wang JL, Cheng Y, Zhang X, Wang H (2013). Extraskeletal Ewing sarcomas in late adolescence and adults: a study of 37 patients. Asian Pac J Cancer Prev.

[20] El Weshi A, Allam A, Ajarim D, Al Dayel F, Pant R, Bazarbashi S (2010). Extraskeletal Ewing’s sarcoma family of tumours in adults: analysis of 57 patients from a single institution. Clin Oncol (R Coll Radiol).

[21] Ahmed SK, Robinson SI, Okuno SH, Rose PS, Issa Laack NN (2014). Adult Ewing sarcoma: survival and local control outcomes in 36 patients with metastatic disease. Am J Clin Oncol.

[22] Seker MM, Kos T, Ozdemir N, Seker A, Aksoy S, Uncu D (2014). Treatment and outcomes of Ewing sarcoma in Turkish adults: a single Centre experience. Asian Pac J Cancer Prev.

[23] Gupta AA, Pappo A, Saunders N, Hopyan S, Ferguson P, Wunder J (2010). Clinical outcome of children and adults with localized Ewing sarcoma: impact of chemotherapy dose and timing of local therapy. Cancer..

[24] Casey DL, Meyers PA, Alektiar KM, Magnan H, Healey JH, Boland PJ (2014). Ewing sarcoma in adults treated with modern radiotherapy techniques. Radiother Oncol.

[25] Lee J, Hoang BH, Ziogas A, Zell JA (2010). Analysis of prognostic factors in Ewing sarcoma using a population-based cancer registry. Cancer..

[26] Paioli A, Luksch R, Fagioli F, Tamburini A, Cesari M, Palmerini E (2014). Chemotherapy-related toxicity in patients with non-metastatic Ewing sarcoma: influence of sex and age. J Chemother.

[27] Diaz-Beveridge R, Lorente D, Torres B, Canete A, Rodrigo E, Bruixola G (2015). Multimodality treatment of pediatric and adult patients with Ewing sarcoma: a single-institution experience. J Pediatr Hematol Oncol.

[28] Biswas B, Bakhshi S (2016). Management of Ewing sarcoma family of tumors: current scenario and unmet need. World J Orthop.

[29] Verrill MW, Judson IR, Harmer CL, Fisher C, Thomas JM, Wiltshaw E (1997). Ewing's sarcoma and primitive neuroectodermal tumor in adults: are they different from Ewing's sarcoma and primitive neuroectodermal tumor in children?. J Clin Oncol.

[30] Fizazi K, Dohollou N, Blay JY, Guerin S, Le Cesne A, Andre F (1998). Ewing's family of tumors in adults: multivariate analysis of survival and long-term results of multimodality therapy in 182 patients. J Clin Oncol.

[31] Lyman GH (2009). Impact of chemotherapy dose intensity on cancer patient outcomes. J Natl Compr Cancer Netw.

[32] Tahover E, Segal A, Isacson R, Rosengarten O, Grenader T, Gips M (2017). Dexrazoxane added to doxorubicin-based adjuvant chemotherapy of breast cancer: a retrospective cohort study with a comparative analysis of toxicity and survival. Anti-Cancer Drugs.

[33] Reichardt P, Tabone MD, Mora J, Morland B, Jones RL (2018). Risk-benefit of dexrazoxane for preventing anthracycline-related cardiotoxicity: re-evaluating the European labeling. Future Oncol.

[34] Lipshultz SE, Scully RE, Lipsitz SR, Sallan SE, Silverman LB, Miller TL (2010). Assessment of dexrazoxane as a cardioprotectant in doxorubicin-treated children with high-risk acute lymphoblastic leukaemia: long-term follow-up of a prospective, randomised, multicentre trial. Lancet Oncol.

[35] Chow EJ, Asselin BL, Schwartz CL, Doody DR, Leisenring WM, Aggarwal S (2015). Late mortality after Dexrazoxane treatment: a report from the Children’s oncology group. J Clin Oncol.

